# Origin and spread of Thoroughbred racehorses inferred from complete mitochondrial genome sequences: Phylogenomic and Bayesian coalescent perspectives

**DOI:** 10.1371/journal.pone.0203917

**Published:** 2018-09-14

**Authors:** Sook Hee Yoon, Wonseok Lee, Hyeonju Ahn, Kelsey Caetano-Anolles, Kyoung-Do Park, Heebal Kim

**Affiliations:** 1 Department of Agricultural Biotechnology and Research Institute for Agriculture and Life Sciences, Seoul National University, Seoul, Republic of Korea; 2 Department of Agricultural Biotechnology, Animal Biotechnology and Research Institute of Agriculture and Life Sciences, Seoul National University, Seoul, Republic of Korea; 3 Department of Agricultural Biotechnology, College of Agriculture and Life Sciences, Seoul National University, Seoul, Republic of Korea; 4 The Animal Molecular Genetics & Breeding Center, Chonbuk National University, Jeonju, Republic of Korea; 5 Institute for Biomedical Sciences, Shinshu University, Nagano, Japan; National Cheng Kung University, TAIWAN

## Abstract

The Thoroughbred horse breed was developed primarily for racing, and has a significant contribution to the qualitative improvement of many other horse breeds. Despite the importance of Thoroughbred racehorses in historical, cultural, and economical viewpoints, there was no temporal and spatial dynamics of them using the mitogenome sequences. To explore this topic, the complete mitochondrial genome sequences of 14 Thoroughbreds and two Przewalski’s horses were determined. These sequences were analyzed together along with 151 previously published horse mitochondrial genomes from a range of breeds across the globe using a Bayesian coalescent approach as well as Bayesian inference and maximum likelihood methods. The racing horses were revealed to have multiple maternal origins and to be closely related to horses from one Asian, two Middle Eastern, and five European breeds. Thoroughbred horse breed was not directly related to the Przewalski’s horse which has been regarded as the closest taxon to the all domestic horses and the only true wild horse species left in the world. Our phylogenomic analyses also supported that there was no apparent correlation between geographic origin or breed and the evolution of global horses. The most recent common ancestor of the Thoroughbreds lived approximately 8,100–111,500 years ago, which was significantly younger than the most recent common ancestor of modern horses (0.7286 My). Bayesian skyline plot revealed that the population expansion of modern horses, including Thoroughbreds, occurred approximately 5,500–11,000 years ago, which coincide with the start of domestication. This is the first phylogenomic study on the Thoroughbred racehorse in association with its spatio-temporal dynamics. The database and genetic history information of Thoroughbred mitogenomes obtained from the present study provide useful information for future horse improvement projects, as well as for the study of horse genomics, conservation, and in association with its geographical distribution.

## Introduction

The Thoroughbred horse, which was developed by English aristocracy during the 17th and 18th centuries, is the world’s best-known racehorse breed [1]. Thoroughbreds can be characterized by several features, including their approximately 165 cm height, a well-chiseled head on a long neck, high withers, deep chest, short back, good hindquarter depth, lean body, and long legs [2]. Due to artificial selection for better racing ability, this breed also shows superior performance characteristics such as large lung volume, high maximum haemoglobin concentration and cardiac output, large muscle mass to body weight ratio, high skeletal muscle mitochondrial density, and oxidative enzyme activity [3–6].

In addition to being an internationally popular spectator sport, horse racing is also a huge industry; its global market value was estimated to be around 115 billion dollars in 2008 [7]. Pedigree is particularly important as the value and potential income of racing horses are dependent on their ancestors’ performance. Improvement and management of the specific animal breed require an understanding of its demography, biogeography, ecology, behavior, genetics, and their interactions. Mitochondrial DNA (mtDNA) has been a very attractive genetic marker for elucidating the domestication history of horses [8–13] due to its relative lack of genetic recombination, maternal inheritance, and the presence of orthologous genes having different evolutionary rates [14]. To date, several studies have investigated the maternal origin of the Thoroughbred horse on the bases of mitochondrial control region (D-loop) [15–18]. Most researchers support that the racehorse breed diverged from multiple maternal origins [15, 16, 18], while Bower et al. [17] proposed that Thoroughbred mares were founded in the British Isles in the 17th and 18th centuries. However, all of these studies used obscure analytical methods and dealt only with D-loop sequences that can generate ambiguous tree topologies due to the high level of recurrent mutations and comparatively short lengths (including a small number of phylogenetically informative sites). Above all, they all didn’t mention time of the most recent common ancestor of the Thoroughbred horses.

In relation to domestic history of the horse, Przewalski’s horse is very important species; this endangered animal is regarded as the only true wild horse species and the closest taxon to the domestic horse. Despite several global studies based genetic information [19–24], horse domestication history with emphasis on phylogenetic relationships of the Thoroughbred and Przewalski’s horses has not been well resolved.

To fully understand the origin and spread of the Thoroughbred racehorses, we determined the complete mitochondrial genome sequences of 14 Thoroughbred racehorses and two Przewalski’s horses. This sequence data was analyzed together with 151 previously published sequences of global horses using phylogenomic and Bayesian coalescent approaches. The objectives of the present study were (1) to study whether the Thoroughbred racehorses have a single maternal origin; (2) to examine the phylogenetic relationships between Thoroughbred horses and other domestic horse breeds; (3) to uncover the phylogenetic relationships of the Thoroughbred and Przewalski’s (the closes taxon to the all domestic horse breeds and the only true wild horse species left in the world) horses; (4) to address the influences of breeds or countries on the heterogeneity of global horses; (5) to estimate the time of the most recent common ancestor of these racehorses; and (6) to assess divergence times of the common female ancestor and changes in the population size of modern horses.

## Materials and methods

### Ethics statement

All animal care and experimental protocols were reviewed and approved by the Institutional Animal Care and Use Committee of Hankyong National University (Ethical Permit Number: HNUAWC-2013-3).

### Horse sample preparation and re-sequencing

Blood samples (10 ml) of 14 Thoroughbreds (Gwacheon racetrack, South Korea) and two Przewalski’s horses (Hustai National Park, Mongolia) were collected from the carotid artery and treated with heparin to prevent coagulation. Genomic DNA was extracted from each blood sample and quality was assessed using fluorescence-based quantification on an 0.6% agarose gel eletrophoresed in pulse field with 200 ng of DNA. Using the TruSeq DNA Sample Prep. Kit (Illumina, San Diego, CA), we constructed the paired-end library (500-bp fragment) according to manufacturer instructions. Briefly, purified genomic DNA fragments of less than 800 bp were blunt-ended with 5’-phosphorylated ends and a 3’-dA overhang. Adaptor-modified ends were added, the ligation product was purified, and a genomic DNA library was constructed. Following this, sequence data was produced using Illumina HiSeq2000.

### Sequence data analysis

Read quality of raw data was assessed using FastQC v 0.10.1 [25]. On the basis of FastQC, we used the Fastx-toolkit to remove sequences with low-quality scores. Using fastq_quality_trimmer of Fastx-toolkit, we trimmed the 3′-end of read base pairs, which had PHRED-scaled quality score of <20. After trimming, reads that were shorter than 80 bp were discarded. The paired-end reads of each sample were aligned to a published mitochondrial genome (GenBank Accession No. X79547) using Bowtie2 v2.0.0-beta6 (http://bowtie-bio.sourceforge.net/bowtie2/index.shtml/) with the no-mixed mode option selected. Summary statistics about used data, alignment rate is summarized in the S2 Table. Picard Tools v 1.7.2 [26], SAMtools v 0.1.18 [27], and GATK v 2.1.8 [28] were conducted for downstream processing and variant calling. Substitution calls were made with GATK UnifiedGenotyper and the following variants were excluded: quality score of <30, three SNPs within a 10-bp window, SNPs in detected InDel mutations, the number or proportion of reads that had mapping quality scores of 0 > 4 or 10%, the number of alternative alleles greater than one (multiallele type), or read depths in the SNP position of <3. Small indels detected by GATK were used for SNP filtering. Number of identified variants for each sample are presented in S3 Table.

### Mitochondrial genome sequence characterization

**Fig 1:**
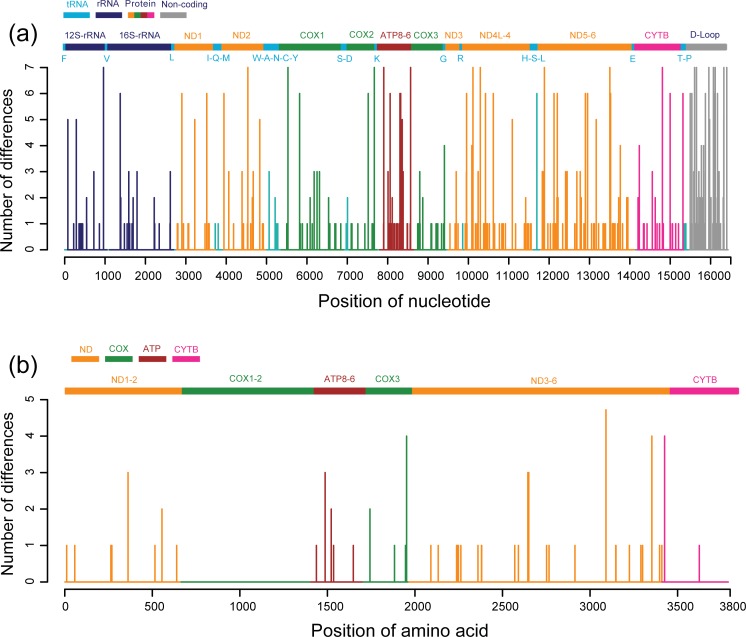
**Plot of the nucleotide (a) and amino acid (b) variations throughout the mitochondrial genomes of 15 Thoroughbred horses.** Number of dissimilarities was calculated as the total number of altered nucleotides at each site compared using the multiple sequence alignment method. Each gene is indicated at the top of each plot. Single letter abbreviations of tRNA genes stand for following full names: F, tRNA-Phenylalanine; V, tRNA-Valine; L, tRNA-Leucine; I, tRNA-Isoleucine; Q, tRNA-Glutamine; M, tRNA-Methionine; W, tRNA-Tryptophan; A, tRNA-Alanine; N, tRNA-Asparagine; C, tRNA-Cysteine; Y, tRNA-Tyrosine; S, tRNA-Serine; D, tRNA-Aspartate; K, tRNA-Lysine; G, tRNA-Glycine; R, tRNA-Arginine; H, tRNA-Histidine; E, tRNA-Glutamate; T, tRNA- Threonine; P, tRNA-Proline.

**Fig 2:**
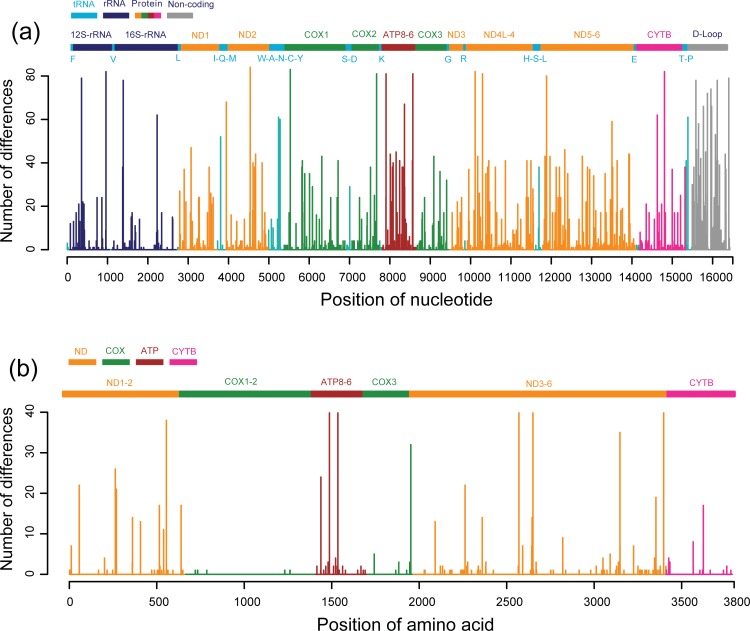
**Plotting the nucleotide (a) and amino acid (b) differences throughout the mitochondrial genomes of 167 modern horses.** Number of differences was estimated as the total number of altered nucleotides at each site compared with the multiple sequence alignment method. Each gene is indicated at the top of each plot. Single letter abbreviations of tRNA genes stand for following full names: F, tRNA-Phenylalanine; V, tRNA-Valine; L, tRNA-Leucine; I, tRNA-Isoleucine; Q, tRNA-Glutamine; M, tRNA-Methionine; W, tRNA-Tryptophan; A, tRNA-Alanine; N, tRNA-Asparagine; C, tRNA-Cysteine; Y, tRNA-Tyrosine; S, tRNA-Serine; D, tRNA-Aspartate; K, tRNA-Lysine; G, tRNA-Glycine; R, tRNA-Arginine; H, tRNA-Histidine; E, tRNA-Glutamate; T, tRNA- Threonine; P, tRNA-Proline.

We analyzed the complete mitochondrial genome sequences of 167 horses (S1 Table). These horses represented 68 breeds, and originated from Asia (n = 45), the Middle East (n = 33), Europe (n = 81), Africa (n = 1), and America (n = 7). Mitochondrial genome sequences were aligned using MAFFT 6.0 [29] and then checked by eye for accuracy and trimmed to minimize missing characters in BIOEDIT 7.053 [30]. The final alignment was 16,432 bp in length, and was comprised of 22 tRNA, 13 protein coding genes, and two rRNA genes. All gene positions quoted here were with respect to a published mitochondrial genome (GenBank accession no. X79547). For both nucleotide and amino acid sequences of each gene, we calculated total sites (including gaps), variable sites, and Ts/Tv ratios using Tree-Puzzle 5.3 [31]. Their sequence identities were estimated using BIOEDIT 7.053 [30], and base frequencies and evolutionary models were generated in Modeltest 3.7 [32] and PAUP 4.0b10 [33]. We subsequently displayed the number of both nucleotide and amino acid variations at each site throughout the mitochondrial genomes (Figs 1 and 2). We first computed the nucleotide differences by counting the number of minor nucleotides at each position across the sequence alignment. Next, we regarded the major nucleotide at each site as the most frequent nucleotide (A, C, G, or T), and the remaining nucleotides were regarded as minor nucleotides. For example, at a specific position of the alignment, if the nucleotide G was major in 125 samples, while A, C, and T were identified as minor nucleotides in the remaining 42 samples, the nucleotide difference was 42. We applied the same rule for amino acid dissimilarity.

For selective pressure analysis, we estimated relative rates of nonsynonymous and synonymous substitutions (ω = dN/dS) across the mitochondrial genomes. Protein coding sequences of genes were verified by comparison with amino acid sequences using Pal2nal [34]. Control files for codeml in PAML4 [35] were set by options as runmode = -2, model = 0, and fix_omega = 0, which let the program estimate dN/dS ratio in pairwise comparison. A dN/dS ratio of <1 means purifying selection, dN/dS = 1 indicates an absence of selection (i.e., neutral evolution), and dN/dS > 1 suggests positive selection.

### Phylogenomic analysis

Phylogenomic analysis was performed using Bayesian inferences (BI) and maximum likelihood (ML) methods. The wild ass (*Equus asinus*) was used as the outgroup [11, 12]. We chose the best-fit model of nucleotide substitutions with the standard Modeltest PAUP block in PAUP 4.0b10 [33] and Akaike’s information criterion (AIC) in Modeltest 3.7 [32]; GTR+I+G was selected as the best fitting evolutionary model. BI analysis was carried out using MrBayes 3.2.5 [36] with the following parameters: nst, 6; rates, gamma; code, vertmt; number of generation, 30,000,000; sample frequency, 500; number of chains, 1; burn-in generation, 25% of the number of generations. Bayesian posterior probability (BPP) values shown on respective internal nodes indicated confidence of the phylogenomic analysis.

In addition to the BI approach, ML analysis was conducted in PHYML 3.0 [37] under the following options: model of nucleotide substitution, GTR; initial tree, BIONJ; nonparametric bootstrap analysis, yes, 500 pseudoreplicates; proportion of invariable sites, estimated; number of substitution rate categories, 6; gamma shape parameter, estimated by program; optimize tree topology, yes.

### Co-estimation of evolutionary rates, time of the most recent common ancestor (tMRCA), and population size changes

To co-estimate the evolutionary rates, times of the most recent common ancestor (tMRCA), and changes in population size, we used the Bayesian Markov Chain Monte Carlo (MCMC) approach which was implemented in BEAST 1.8.2 [38]. For the calibration point, we assumed a bifurcation time of 2 My between the horse and donkey (assuming a 95% interval of 1.56–2.44 My), which is the approximate date of the first fossil record of caballoid horses [8, 39]. Here, GTR+I+G and nst = 6 and rates = gamma were used as the best fit evolutionary model and likelihood setting, respectively, which were derived from AIC in Modeltest 3.7 [32]. Subsequently, we employed both strict and relaxed (uncorrelated exponential and uncorrelated lognormal) molecular clocks [38] with six different demographic models (constant size, exponential growth, expansion growth, logistic growth, Bayesian skyline, and Yule process). Using the Bayes factor test (log_10_ Bayes Factors > 2 in all cases) based on the harmonic mean of the marginal log-likelihoods [40], the relaxed uncorrelated lognormal clock and Yule process model were selected as showing the best fit for the horse data matrix. The effective population size changes over time were evaluated using the Bayesian skyline plot (BSP) analysis. The data sets were run for 30,000,000 generations to ensure convergence of all parameters (ESSs >200); the first 10% of samples for each chain were discarded as burnin. The resulting convergence was analyzed using Tracer 1.5 (http://beast.bio.ed.ac.uk/Tracer) and the statistical uncertainties were summarized in the 95% highest probability density (HPD) intervals. Maximum Clade Credibility (MCC) phylogenetic trees were summarized using the TreeAnnotator program in the BEAST package, and visualized using FigTree 1.4.2 software [41].

## Results

### Mitochondrial genome sequences from both Thoroughbred and Przewalski’s horses

The complete mitochondrial genomes of the 14 Thoroughbreds and two Przewalski’s horses were sequenced in the present study (GenBank accession nos., KT221830 –KT221845) (S1 Table). The genome sizes varied from 16,656 bp (ThorK02) to 16,664 bp (ThorK06) in length, which are well within the range reported for other completely sequenced horses (usually ranging from 16,646 bp to 16,666 bp) (e.g. [11, 12]).

**Fig 3:**
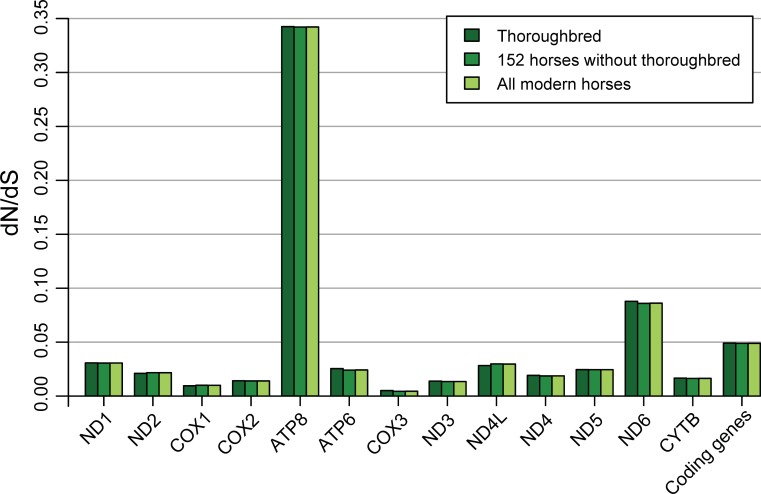
Pairwise dN/dS (ω) values of the mitochondrial genomes of 167 modern horses. By comparing the relative rates of nonsynonymous and synonymous substitutions in 13 protein coding genes, we discovered that the ATP8 gene in both Thoroughbred racehorses and other modern horses has the highest level of adaptive variation.

For all Thoroughbreds (14 new and one published sequences), the configuration of the complete mitochondrial genomes and their individual gene sequences are summarized in Table 1 and Fig 1. The nucleotide sequence identities of the 15 Thoroughbred samples ranged from 99.8% to 100% (average, 99.6%), which corresponds to 99.6–100% (average, 99.8%) identity at the amino acid level. The Ts/Tv ratio estimated from our Thoroughbred horse data set was 29.44. Of the 17 individual genomic regions, NADH6 was the most variable (average sequence identities of 98.7% and 99.6% for nucleotides and amino acids, respectively), whereas 22tRNAs were the most conserved (average nucleotide similarities of 99.9%). We plotted the nucleotide and amino acid differences at each site throughout the mitochondrial genome alignment (Fig 1). Here, higher sequence diversity for nucleotides was presented in the D-loop region and lower amino acid variation was detected in COX1-3 regions. The mean nonsynonymous/synonymous substitution ratio (**ω** = dN/dS) values for each component gene of the racing horses were lower than 1 (Table 1 and Fig 3), which suggests that Thoroughbred mitochondrial genomes were under purifying selection. The highest dN/dS ratio was observed in ATPase8 (0.343), whilst the lowest one was found in COX1 (0.010). We additionally characterized mitochondrial genomes of 167 global horses to compare with those from racing horses. Estimates of the all modern horse mitochondrial genomes are very similar with those from Thoroughbreds (Table 2, Figs 2 and 3).

**Table 1:** Summary of mitochondrial genome regions of 15 thoroughbred horses used in this study.

Genomic region	Total sites including gaps, nt/ aa	Variable sites (%),/ aa	Sequence identities (%),(average), nt/ aa	*Ts/Tv ratio	Base frequencies,A, C, G (%)	**ω value (*dN/dS*)
12S rRNA	977/ -	15(1.5%)/ -	99.2–100.0(99.7)/ -	29.67	36.6, 24.1, 17.0	-
16S rRNA	1,581/ -	19(1.2%)/ -	99.4-100(99.8)/ -	19.46	37.8, 22.2, 16.9	-
NADH1	954/ 318	14(1.5%)/ 4(1.3%)	99.3-100(99.7)/ 98.7-100(98.3)	29.67	36.1, 24.1, 17.4	0.031
NADH2	1,038/ 346	15(1.4%)/ 4(1.2%)	99.2-100(99.7)/ 99.1-100(99.8)	29.68	36.8, 23.8, 17.5	0.021
COX1	1,542/ 514	18(1.2%)/ 0(0.0%)	99.4-100(99.8)/100-100(100)	18.48	37.8, 22.4, 16.9	0.010
COX2	681/ 227	8(1.2%)/ 0(0.0%)	99.0-100(99.7)/ 100-100(100)	28.60	35.8, 24.2, 16.4	0.014
ATPase8	201/ 67	1(0.5%)/ 1(1.5%)	99.5-100(99.8)/ 98.5-100(99.8)	29.67	34.8, 20.2, 17.4	0.343
ATPase6	678/ 226	12(1.8%)/ 4(1.8%)	99.0-100(99.7)/ 98.7-100(99.6)	29.67	35.8, 24.3, 16.5	0.026
COX3	783/ 261	13(1.7%)/ 4(1.5%)	99.1-100(99.7)/ 98.8-100(99.7)	29.67	36.0, 24.4, 16.7	0.005
NADH3	345/ 115	3(0.9%)/ 0(0.0%)	99.1-100(99.7)/ 100-100(100)	29.66	34.2, 22.3, 18.3	0.014
NADH4L	294/ 98	3(1%)/ 2(2.0%)	99.0-100(99.6)/ 97.9-100(99.7)	29.67	35.4, 21.7, 17.3	0.028
NADH4	1,377/ 459	16(1.2%)/ 9(2.0%)	99.3-100(99.7)/ 99.6-100(99.9)	29.67	37.7, 23.1, 16.8	0.019
NADH5	1,812/ 604	24(1.3%)/ 9(1.5%)	99.5-100(99.7)/ 99.3-100(99.7)	13.08	38.1, 22.2, 16.3	0.025
NADH6	528/ 175	26(4.9%)/ 5(2.9%)	97.9-100(98.7)/ 98.9-100(99.6)	14.05	25.7, 13.1, 28.3	0.088
Cytb	1,140/ 379	15(1.3%)/ 2(0.5%)	99.3-100(99.7)/ 99.5-100(99.9)	29.67	37.2, 23.7, 17.1	0.017
Control region	962/ -	15(1.6%)/ -	99.2-100(99.7)/ -	29.67	36.2, 24.2, 17.3	-
13 protein coding genes	11,373/ 3,789	196(1.7%)/ 44(1.2%)	99.2-100(99.6)/ 99.6-100(99.8)	29.56	30.2, 29.3, 13.3	0.049
22 tRNAs	1,520/ -	13(0.9%)/ -	99.7-100(99.9)/ -	29.67	32.7, 18.5, 19.4	-
Overall	16,432 -	299(1.8%)/ -	99.8-100(99.6)/ -	29.44	32.4, 28.4, 13.2	-

*Ts/Tv ratio = transition versus transversion ratio

*ω (dN/dS) value = relative rates of nonsynonymous and synonymous substitutions

**Table 2:** Summary of mitochondrial genome regions of 167 modern horses used in this study.

Genomic region	Total sites including gaps, nt/ aa	Variable sites (%), nt/ aa	Sequence identities (%), (average), nt/ aa	*Ts/Tv ratio	Base frequencies, A, C, G	Evolutionary model	Nst	Rates	**ω value (*dN/dS*)
12S rRNA	977/ -	50(5.1%)/ -	98.7–100.0(99.7)/ -	9.21	36.7, 24.2, 17.0	TIM+I+G	6	gamma	-
16S rRNA	1,581/ -	65(4.1%)/ -	99.3-100(99.7)/ -	10.04	37.8, 22.2, 16.9	TrN+I+G	6	gamma	-
NADH1	954/ 318	47(4.9%)/ 10(3.1%)	98.7-100(99.7)/ 98.4-100(99.7)	8.10	36.1, 24.2, 17.5	GTR+G	6	gamma	0.031
NADH2	1,038/ 346	50(4.8%)/ 19(5.5%)	98.7-100(99.6)/ 98.5-100(99.7)	9.14	36.8, 23.8, 17.6	HKY+I	2	equal	0.022
COX1	1,542/ 514	62(4.0%)/ 4(0.8%)	99.1-100(99.7)/ 99.8-100(99.9)	9.65	37.8, 22.4, 16.9	HKY+I	2	equal	0.010
COX2	681/ 227	37(5.4%)/ 4(1.8%)	98.5-100(99.7)/ 99.1-100(99.9)	5.64	35.9, 24.3, 16.5	HKY+I	2	equal	0.014
ATPase8	201/ 67	12(6.0%)/ 5(7.5%)	98.5-100(99.8)/ 97.0-100(99.5)	3.01	34.9, 20.4, 17.4	K81uf	6	equal	0.342
ATPase6	678/ 226	37(5.5%)/ 14(6.2%)	98.5-100(99.7)/ 97.8-100(99.5)	5.64	35.9, 24.4, 16.6	TrN+G	6	gamma	0.024
COX3	783/ 261	41(5.2%)/ 7(2.7%)	98.6-100(99.7)/ 98.8-100(99.8)	6.93	36.1, 24.5, 16.8	HKY+I	2	equal	0.005
NADH3	345/ 115	20(5.8%)/ 2(1.7%)	98.5-100(99.7)/ 98.3-100(99.9)	4.91	34.2, 22.6, 18.2	K81uf	6	equal	0.014
NADH4L	294/ 98	22(5.8%)/ 4(4.1%)	98.3-100(99.7)/ 97.9-100(99.8)	3.95	35.4, 22.0, 17.3	K81uf+G	6	gamma	0.030
NADH4	1,377/ 459	54(3.9%)/ 22(4.8%)	99.1-100(99.7)/ 99.1-100(99.8)	9.88	37.8, 23.1, 16.8	K81uf+I	6	equal	0.019
NADH5	1,812/ 604	73(4.0%)/ 26(4.3%)	99.2-100(99.7)/ 98.8-100(99.7)	9.7	38.1, 22.2, 16.3	TIM+I	6	equal	0.025
NADH6	528/ 175	49(9.3%)/ 20(11.4%)	97.1-100(98.7)/ 97.1-100(99.6)	8.61	25.6, 13.1, 28.3	TVM+G	6	gamma	0.086
Cytb	1,140/ 379	51(4.5%)/ 11(2.9%)	98.5-100(98.9)/ 98.9-100(99.9)	9.37	37.2, 23.8, 17.2	TrN+I	6	equal	0.016
Control region	963/ -	49(5.1%)/ -	98.6-100(99.6)/ -	9.13	36.3, 24.2, 17.3	GTR+I+G	6	gamma	-
13 protein coding genes	11,373/ 3,789	589(5.2%)/ 148(3.9%)	98.9-100(99.5)/ 99.5-100(99.8)	26.20	30.2, 29.3, 13.3	TIM+I+G	6	gamma	0.049
22 tRNAs	1,520/ -	46(3%)/ -	99.3-100(99.8)/ -	3.46	32.7, 18.5, 19.5	TrN+I+G	6	gamma	-
Overall	16,432/ -	845(5.1%)/ -	99.0-100(99.5)/ -	18.29	32.5, 28.4, 13.2	GTR+I+G	6	gamma	-

*Ts/Tv ratio = transition versus transversion ratio

**ω (dN/dS) value = relative rates of nonsynonymous and synonymous substitutions

### Phylogenomic analyses

The 16,432 bp long (including gaps) mitochondrial genome alignment showed high variation with 845 polymorphic sites (5.1% of total number of sites). The nucleotide sequence identities among the 167 modern horses ranged from 99.0% to 100%. Configuration of the mitochondrial genomes and their 38 individual regions for the 167 horses are reported in Table 2, Figs 2 and 3.

**Fig 4:**
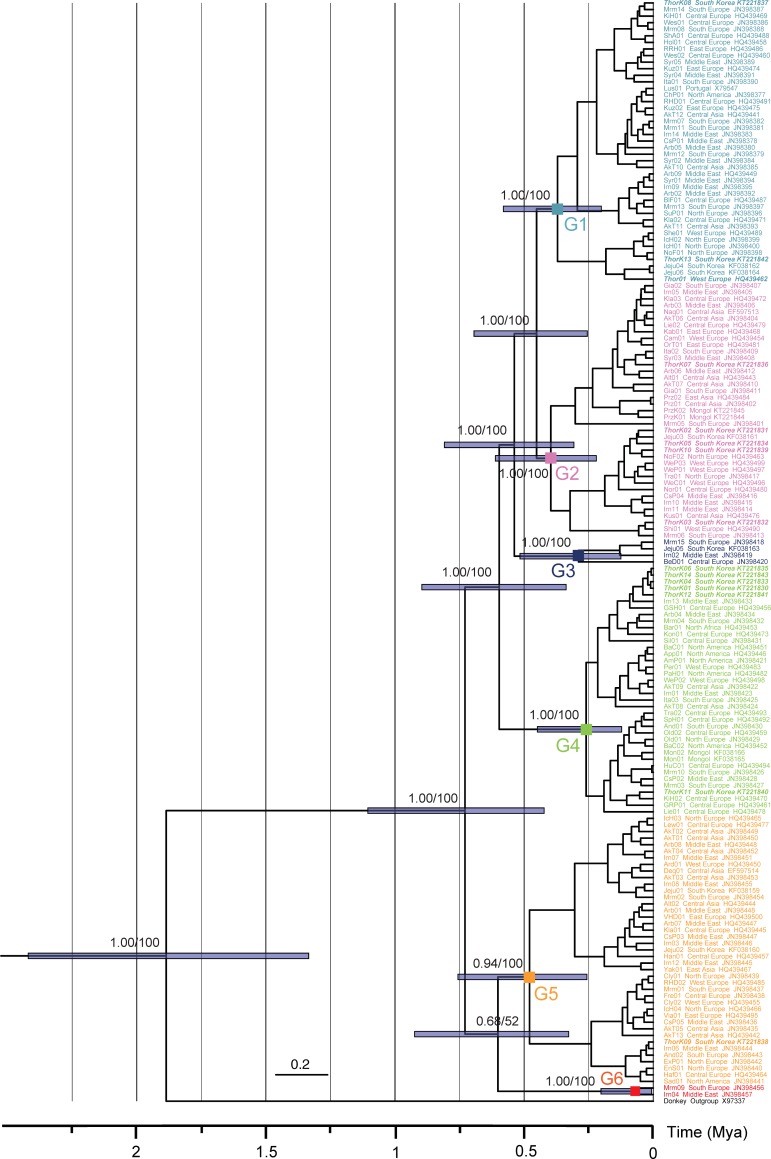
Bayesian maximum clade credibility phylogenomic tree on the ground of the mitochondrial genome sequences of 167 modern horses. The data set (16,432 base pairs) was also analyzed phylogenetically using Bayesian inference (BI) and maximum likelihood (ML) methods which showed the same topologies. 95% Highest Posterior Density of node heights are shown by blue bars. Groups are marked by a “G”. Numbers at the nodes represent (left to right): posterior probabilities (≥0.80) for the BI tree and bootstrap values (≥70%) for the ML tree. The racing horses were revealed to have multiple maternal origins and to be closely related to horses from one Asian, two Middle Eastern, and five European breeds. Results of phylogenomic analyses also uncovered no apparent association between geographic origin or breed and heterogeneity of global horses. The most recent common ancestor of the Thoroughbreds lived approximately 8,100–111,500 years ago, which was significantly younger than the most recent common ancestor of modern horses (0.7286 My).

**Fig 5:**
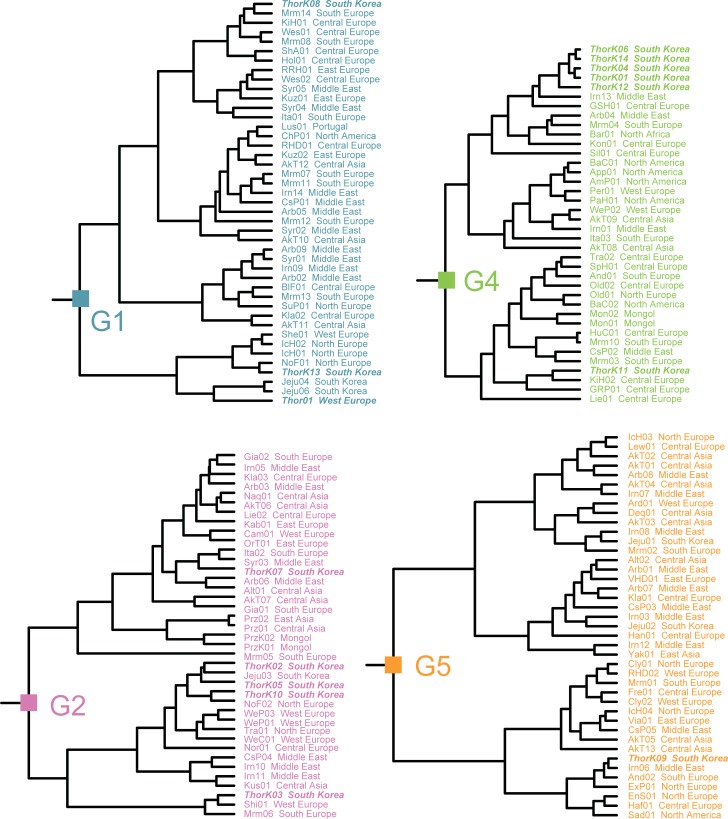
Distribution of Thoroughbred horses within the Bayesian maximum clade credibility phylogenetic tree derived from the complete mitochondrial genome sequences of 167 global horses. Thoroughbred horse samples are shown in italic and bold type.

The matrilineal line of the modern horses was illustrated as a maximum clade credibility tree (Fig 4), which was supported by tree topologies resulting from both Bayesian inferences (BI) and maximum likelihood (ML) methods. The 167 global horses were classified into one of six major groups with high confidence for each node. Here, Thoroughbred breed was not monophyletic and the 15 specimens were divided into four different clades (Figs 4 and 5): Group 1 (Thor01, ThorK08, and ThorK13), Group 2 (ThorK02, ThorK03, ThorK05 ThorK07, and ThorK10), Group 4 (ThorK01, ThorK04, ThorK06, ThorK11, ThorK12, and ThorK14), and Group 5 (ThorK09). Within Group 1, racing horses were closely related to one of the two different European and one Asian breeds: Maremmano (Mrm14), Norwegian Fjord (NoF01), and Jeju (Jeju04 and Jeju06). The Thoroughbred individuals of Group 2 were clustered with breeds from three different European, one Middle East, and one Asian breeds: Shire (Shi01), Italian (Ita02), Norwegian Fjord (NoF02), Syrian (Syr03), and Jeju (Jeju03). The Thoroughbreds of Group 4 were linked to Iranian breed (Irn13) from the Middle East and Kinsky horse breed (KiH02) from Central Europe, while Group 5 specimen was clustered with the Iranian breed (Irn06) from the Middle East.

We focused on the genetic relationships between the Thoroughbred and Przewalski’s horses. As mentioned above, our phylogenomic trees showed that this racing horse breed was not directly related to the Przewalski’s horse. Monophyletic Przewalski’s horses appeared as members of Group 2, and were linked to a clade consisting of 14 different domestic breeds derived from Asia, the Middle East, and Europe: Akhal-Teke (AkT06 and AkT07), Altai (Alt01), Arabian (Arb03 and Arb06), Camargue (Cam01), Giara (Gia01 and Gia02), Iranian (Irn05), Italian (Ita02), Kabardin (Kab01), Kladruber (Kla03), Liebenthaler (Lie02), Naqu (Naq01), Orlov Trotter (OrT01), Thoroughbred (ThorK07), and Syrian (Syr03).

Subsequently, we further studied on the influences of breeds or countries and heterogeneity of global horses. Within the global horse trees, there was no geographic grouping within the six groups with a mixture of breeds from different geographic areas grouping into the same clade (Fig 4; Table 3). The individuals in Group 1 (n = 43) were collected from four different regions: Asia (n = 7), the Middle East (n = 10), Europe (n = 25), and America (n = 1). Their breeds were a mixture of 24 different ones. Horses of Group 2 (n = 39) originated from Asia (n = 15), Middle East (n = 7), and Europe (n = 17), and were included in 25 different breeds. Subsequently, Group 3 (n = 4) was composed of Asian (n = 1), Middle Eastern (n = 1), and European (n = 2) isolates, and a mixture of four different breeds. Specimens of Group 4 (n = 38) were selected from Asia (n = 10), the Middle East (n = 4), and Europe (n = 18), as well as Africa (n = 1) and America (n = 5). And they comprised of 26 different breeds. Group 5 (n = 41) members were sampled from Asia (n = 12), the Middle East (n = 10), Europe (n = 18), and America (n = 1), and were a mixture of 25 different breeds. Group 6 was grouped with two specimens of two breeds from the Middle East and Europe (Fig 3). Accordingly, the branching pattern of the modern horse tree did not appear to be influenced by the geographic origin of breeds.

**Table 3:** Summary of the six horse groups.

Group	No. of horses	Kinds of breeds	Geographic regions	tMRCA (Mya)
1	43	AkT, Arb, BlF, ChP, CsP, Hol, IcH, Irn, Ita, Jeju, KiH, Kla, Kuz, Lus, Mrm, NoF, RHD, RRH, ShA, She, SuP, Syr, ***Thor*,** Wes (n = 24)	Asia, Middle East, Europe, America	0.3704
2	39	AkT, Alt, Arb, Cam, CsP, Gia, Irn, Ita, Jeju, Kab, Kla, Kus, Lie, Mrm, Naq, NoF, Nor, OrT, Prz, Shi, Syr, ***Thor*,** Tra, WeC, WeP (n = 25)	Asia, Middle East, Europe	0.3964
3	4	BeD, Irn, Jeju, Mrm (n = 4)	Asia, Middle East, Europe	0.2892
4	38	AkT, AmP, And, App, Arb, BaC, Bar, CsP, GRP, GSH, HuC, Irn, Ita, KiH, Kon, Lie, Mon, Mrm, Old, PaH, Per, Sil, SpH, ***Thor*,** Tra, WeP (n = 26)	Asia, Middle East, Europe, Africa, America	0.2599
5	41	AkT, Alt, And, Arb, Ard, Cly, CsP, Deq, EnS, ExP, Fre, Haf, Han, IcH, Irn, Jeju, Kla, Lew, Mrm, RHD, Sad, ***Thor*,** VHD, Via, Yak (n = 25)	Asia, Middle East, Europe, America	0.4789
6	2	Mrm, Irn (n = 2)	Middle East, Europe	0.07

### Divergence times, subtitution rates, and population size changes

Results of analyses performed by BEAST 1.8.2 revealed that the relaxed uncorrelated lognormal clock and Yule process model was the best-fit for simulating evolution of 167 horse mitochondrial genomes. Under this model, estimates for Thoroughbred horses ranged from 8,100 years (ThorK14) to 111,500 years (Thor01) (S4 Table). According to the BMCC tree, the most recent common ancestor of the modern horses existed approximately 0.7286 My ago (95% HPD = 0.4225–1.1051); Group 5 emerged first (0.4789 My ago, 95% HPD = 0.2569–0.7563), followed by sequential segregation of Group 2 (0.3964 My ago, 95% HPD = 0.2202–0.6114), Group 1 (0.3704 My ago, 95% HPD = 0.2009–0.5805), Group 3 (0.2892 My ago, 95% HPD = 0.1253–0.5161), Group 4 (0.2599 My ago, 95% HPD = 0.1224–0.4486), and Group 6 (0.07 My ago, 95% HPD = 0.007–1.2024) (Fig 4 and Table 3). The evolutionary rate of the 167 modern horses was estimated to be 1.469 × 10^−2^ substitutions/site/My (95% HPD = 8.8143 × 10−3–2.1600 × 10^−2^).

**Fig 6:**
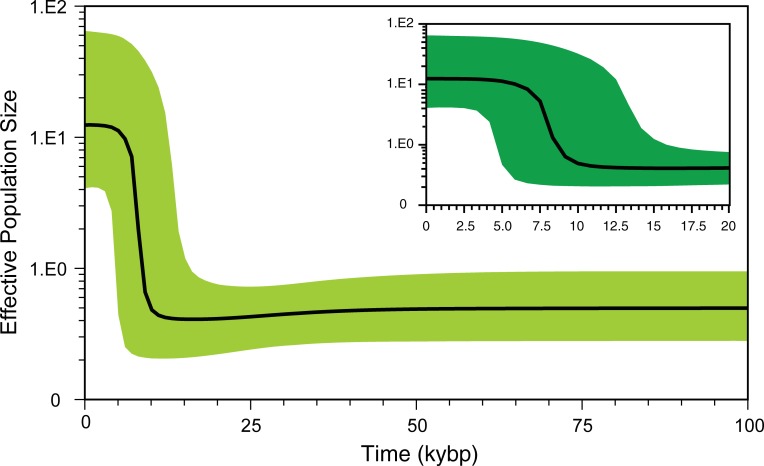
Bayesian skyline plot (BSP) based on mitochondrial genome sequences from 167 modern horses. The dark line in the BSP represents the estimated effective population size through time. The green area represents the 95% highest posterior density confidence intervals for this estimate.

In the constructed Bayesian skyline plot, modern horses remained at an almost constant population size until about 11,000 years ago, when they experienced a rapid expansion of population until about 5,500 years ago when domestication may have occurred (Fig 6).

## Discussion

Two different hypotheses exist on the domestication mode of Thoroughbred horses: the single origin hypothesis and the multiple origin scenario. These controversial hypotheses have important implications on genetic variation in maternally inherited mtDNA. The single origin hypothesis implies that mitochondrial diversity should be limited to a few ancestral lineages and then be accumulated in their progenies subsequently by mutation, while in the multiple origin scenario the mitochondrial dissimilarity of the horses is suggested to be greater than that of a single wild population. The results of the present study support the multiple origin hypothesis. The average nucleotide sequence variability among the 15 Thoroughbred mitochondrial genomes was 0.4%; this estimate is very similar to that (0.5%) of global horses. Furthermore, as expected, the Ts/Tv ratio of the Thoroughbred mitochondrial genome data set was 29.44; this estimate is much higher than 18.29 of the global horse sequences. These results support that the Ts/Tv ratio is highly influenced by the level of phylogenetic divergence. Particularly, in all of our phylogenomic trees, Thoroughbred horses were classified into four different groups with high robustness. Previous works based on archival records [42] and mitochondrial D-loop sequences [15, 16, 18] were also in line with the multiple origin scenario. Unlike these supporters to multiple origin, however, several scientists support a single origin hypothesis. Wallace [43] proposed that the Thoroughbred foundation mares were natives of the British Isles, while Wentworth [44] suggested that their female origin was of imported Arab mares; both researchers performed their studies using historical records. Bower et al. [17] asserted that Thoroughbred mares were founded in the British Isles on the basis of mitochondrial D-loop sequences.

In terms of the genetic relationship of Thoroughbred horses and the global horses, our results of phylogenomic analysis revealed that the Thoroughbreds were closely related to some horses from eight breeds: one (Jeju) from Asia, two (Syrian and Iranian) from Middle East, and five (Maremmano, Norwegian Fjord, Shire, Italian, and Kinsky) from Europe. However, there were contradicting studies on this topic, on the basis of histological records [42–44] and mitochondrial D-loop data [17].

Next, present study focused on relationships between domestic horses and the Przewalksi’s horses. Endangered Przewalski’s horse is regarded as the closest taxon to the domestic horse and the only true wild horse species. Several phylogeny workers such as Ryder [22] and Orlando [23] claimed that domestic horses were derived from Przewalski’s horse based on mitochondrial control region and genomic sequences, respectively. Wade et al. [24] suggested that Przewalski’s horse evolved from domestic horses by means of autosomal single-nucleotide polymorphisms (SNPs). However, the findings of the present study do not support their viewpoints; Monophyletic Przewalski’s horse was not a direct progenitor of domestic horses; domestic horses did not diverge from Przewalski’s horse, or vice versa. This finding was consistent with previous studies based on autosomal and X chromosome introns [19], partial control region [20], and both mitochondrial and partial nuclear genome sequences [21]. Our analyses also revealed that this Thoroughbred horse breed was not directly linked to the wild horse. It is interesting to study the horse domestication history with an emphasis on the phylogenetic relationships of the Thoroughbred and Przewalski’s horses; there was no investigation of this topic. Understanding the evolutionary relationship of Thoroughbreds and Przewalksi’s horses is incredibly important for conservation and breeding strategies.

Additionally, our phylogenomic analysis revealed that no apparent correlation between breed or geographic location and evolution of global horses. Within our phylogenomic trees, horses from different breed or geographic location were more closely related one another rather than those from the same breeds or regions, respectively. The configuration of mixtures of mitochondrial genome sequences was also in concordance with the views of other authors [8, 9, 11–13] and maybe largely due to their rapid motion and international trade in livestock. On contrast to our results, however, several authors postulated the geographic partitioning of horses based on mitochondrial [45–47] and microsatellite [48] sequences. They suggested the important factor of geographic structure of horse is geographic isolation by Silk Road, high mountain, and etc.

We carried out a Bayesian coalescent approach using extended mitochondrial genome sequences from 167 horses in order to further assess the timescale of horse domestication. Here, we first calculated the time of the most recent common ancestor of Thoroughbred horses. Our analysis revealed the age of the most recent common ancestor of the racing horse to be around 8,100–111,500 years old. This estimate is much younger than that of the most recent common ancestor of the global horses, which has been estimated at 0.7286 Mys old.

On the domestication time of modern horses, there have been several publications derived from both archaeological [49–51] and molecular [11–12, 23, 48] evidences. D’Andrade [49] reported that the origin of domestic horses was around 4,000 years ago. Ludwig et al. [50] stated the domestication time to be about 5,000 years ago, while Anthony [51] noted that horse rearing by humans may have occurred approximately 6,000 years ago. Subsequently, on the basis of mitochondrial genome sequences, Lippold et al. [11] and Achilli et al. [12] postulated domestication time to be about 6,000–8,000 and 6,000–7,000 years ago, respectively. Warmuth [48] dated domestication time to 5,500 years ago based on autosomal genotype data, while Orlando et al. [23] claimed that Przewalski’s and domestic horse populations diverged 38,000–72,000 years ago based on analysis of genome sequences. In contrast to the previous hypothesized date of horse domestication, the results of our Bayesian skyline plot (BSP) analysis depict a rapid expansion of the horse population approximately 5,500–11,000 years ago, which coincides with the start of domestication.

Information about the genetic diversity of Thoroughbred mitogenomes can improve our understanding of its emergence. Our findings indicated that ATPase 8 and NADH6 of the racing horses are more variable than other genes. ATP8 showed the highest dN/dS ratio, while ND6 showed the most variable sequence (the lowest sequence identity) and the second highest dN/dS ratio. The dN/dS estimation is used as an estimate of whether or not the subject has undergone adaptive evolution to gain advantages due to amino acid changes [52]. In other words, it is a comparison of the ratio of non-synonymous and synonymous substitutions in the substitution that occurred, so the high dN/dS ratio means that the non-synonymous substitution (amino acid change) occurred at a higher rate than the synonymous substitution (no amino acid change). This is slightly different from sequence identity (%), which can be used to examine sequence variability. In a study of the mitochondrial genome evolution of Chinese domestic horses, the researchers have shown that ATPase8 and NADH6 exhibit higher Ka/Ks ratios [53]. The results are in line with the dN/dS analysis results of our study (Fig 3). In the same paper, researchers said that ATPase8 and NADH6 together with ATPase6 and Cytb are genes that contribute to the OXPHOS system (the metabolic pathway releasing energy using ATP). These gene functions also can be a reason that ATPase8 and NADH6 show a strong dN/dS ratio (strong directional selection), and that NADH6 is the most variable sequence.

Thoroughbreds are the most well-known breed for horse racing, and have been artificially selected for structural and functional adaptations that contribute to athletic performance phenotypes. Improvement and management of the specific animal breed need an understanding of its domestication history, demography, biogeography, behavior, genetics, and their interactions, which is obtained from molecular phylogenetic investigation. The database and genetic history information of Thoroughbred mitogenomes gathered from the present study provide useful information for future horse improvement projects as well as for studies investigating horse genomics, conservation, and geographical distribution. This is the first study on the spatio-temporal dynamics of the Thoroughbred racehorse.

## Supporting information

S1 Table 167 horse isolates (68 breeds) used in this study. (DOC)

S2 Table Summary statistics for number of reads used in this study and alignment rate using SAMtools. (DOCX)

S3 Table Number of variants (SNPs and InDels) identified using GATK. (DOCX)

S4 Table tMRCA of Thoroughbred horses. (DOCX)

## References

[1] Cassidy R. The sport of kings: kinship, class and thoroughbred breeding in Newmarket. Cambridge, UK: Cambridge University Press; 2002.

[2] Montgomery ES. The thoroughbred. New York, NY: Arco Publishing; 1980.

[3] Essen-Gustavsson B, Lindholm A. Muscle fibre characteristics of active and inactive standardbred horses. Equine Vet J. 1985; 17: 434–438. 4076157 10.1111/j.2042-3306.1985.tb02549.x

[4] Poso AR, Essengustavsson B, Persson SGB. Metabolic response to standardized exercise test in standard-bred trotters with red-cell hypervolemia. Equine Vet J. 1993; 25: 527–531. 8276001 10.1111/j.2042-3306.1993.tb03007.x

[5] Hyyppa S, Rasanen LA, Poso AR. Resynthesis of glycogen in skeletal muscle from standardbred trotters after repeated bouts of exercise. Am J Vet Res. 1997; 58: 162–166. 9028482

[6] Hinchcliff KW, Geor RJ. The horse as an athlete: a physiological overview. Equine exercise physiology: the science of exercise in the athletic horse. Edinburgh; New York: Saunders/Elsevier; 2008 pp ix, 463.

[7] Global betting stable, but some countries suffer recession. Horseracingintfed.com. 2009-10-07.

[8] Vilà C, Leonard JA, Götherström A, Marklund S, Sandberg K, Lidén K, et al Widespread origins of domestic horse lineages. Science. 2001; 291: 474–477. 10.1126/science.291.5503.474 11161199

[9] Jansen T, Forster P, Levine MA, Oelke H, Hurles M, Renfrew C, et al Mitochondrial DNA and the origins of the domestic horse. Proc Natl Acad Sci USA. 2002; 99: 10905–10910. 10.1073/pnas.152330099 12130666 PMC125071

[10] Cieslak M, Pruvost M, Benecke N, Hofreiter M, Morales A, Reissmann M, et al Origin and history of mitochondrial DNA lineages in domestic horses. PLoS ONE. 2010; 5: e15311 10.1371/journal.pone.0015311 21187961 PMC3004868

[11] Lippold S, Matzke NJ, Reissmann M, Hofreiter M. Whole mitochondrial genome sequencing of domestic horses reveals incorporation of extensive wild horse diversity during domestication. BMC Evol Biol. 2011; 11: 328 10.1186/1471-2148-11-328 22082251 PMC3247663

[12] Achilli A, Olivieri A, Soares P, Lancioni H, Kashani BH, Perego UA, et al Mitochondrial genomes from modern horses reveal the major haplogroups that underwent domestication. Proc Natl Acad Sci USA. 2012; 109: 2449–2454. 10.1073/pnas.1111637109 22308342 PMC3289334

[13] Der Sarkissian C, Ermini DL, Schubert M, Yang MA, Librado P, Fumagalli M, et al Evolutionary Genomics and Conservation of the Endangered Przewalski's Horse. Curr Biol. 2015; 25: 2577–2583. 10.1016/j.cub.2015.08.032 26412128 PMC5104162

[14] Saccone C, Giorgi CD, Gissi C, Pesole G, Reyes A. Evolutionary genomics in Metazoa: the mitochondrial DNA as a model system. Gene. 1999; 238: 195–209. 10570997 10.1016/s0378-1119(99)00270-x

[15] Hill EW, Bradley DG, Al-Barody M, Ertugrul O, Splan RK, Zakharov I, et al History and integrity of thoroughbred dam lines revealed in equine mtDNA variation. Anim Genet. 2002; 33: 287–294. 12139508 10.1046/j.1365-2052.2002.00870.x

[16] Harrison SP, Turrion-Gomez JL. Mitochondrial DNA: an important female contribution to thoroughbred racehorse performance. Mitochondrion. 2006; 6: 53–63. 10.1016/j.mito.2006.01.002 16516561

[17] Bower M, Campana M, Whitten M, Edwards CJ, Jones H, Barrett E, et al The cosmopolitan maternal heritage of the Thoroughbred racehorse breed shows a significant contribution from British and Irish native mares. Biol Lett. 2011; 7: 316–320. 10.1098/rsbl.2010.0800 20926431 PMC3061175

[18] Bower MA, Whitten M, Nisbet RER, Spencer M, Dominy KM, Murphy AM, et al Thoroughbred racehorse mitochondrial DNA demonstrates closer than expected links between maternal genetic history and pedigree records. J. Anim. Breed. Genet. 2013; 130: 227–235. 10.1111/j.1439-0388.2012.01018.x 23679948

[19] Lau AN, Peng L, Goto H, Chemnick L, Ryder OA, Makova KD. Horse domestication and conservation genetics of Przewalski’s horse inferred from sex chromosomal and autosomal sequences. Mol Biol Evol 2009; 26: 199–208. 10.1093/molbev/msn239 18931383

[20] Ishida N, Oyunsuren T, Mashima S, Mukoyama H, Saitou N. Mitochondrial DNA sequences of various species of the genus *Equus* with special reference to the phylogenetic relationship between Przewalskii's wild horse and domestic horse. J Mol Evol. 1995; 41: 180–188. 7666447 10.1007/BF00170671

[21] Goto H, Ryder OA, Fisher AR, Schultz B, Kosakovsky SL. A Massively parallel sequencing approach uncovers ancient origins and high genetic variability of endangered Przewalski's horses. Genome Biol Evol. 2011; 3: 1096–1106. 10.1093/gbe/evr067 21803766 PMC3194890

[22] Ryder OA. Genetic studies of Przewalski’s horses and their impact on conservation In Przewalski’s horse: The History and Biology of an Endangered Species. Edited by Boyd L, Houpt KA, Albany (NY): The State University of New York Press 1994: 75–92.

[23] Orlando L, Ginolhac A, Zhang G, Froese D, Albrechtsen A. Recalibrating *Equus* evolution using the genome sequence of an early Middle Pleistocene horse. Nature. 2013; 499: 74–78. 10.1038/nature12323 23803765

[24] Wade CM, Giulotto E, Sigurdsson S, Zoli M, Gnerre S, Imsland F, et al Genome sequence, comparative analysis, and population genetics of the domestic horse. Science. 2009; 326: 865–867. 10.1126/science.1178158 19892987 PMC3785132

[25] FastOC project. at http://www.bioinformatics.babraham.ac.uk/projects/fastqc/

[26] Picard project. at http://sourceforge.net/projects/picard/.

[27] Li H, Handsaker B, Wysoker A, Fennell T, Ruan J, Homer N. 1000 Genome project data processing subgroup. The sequence alignment/map format and SAMtools. Bioinformatics. 2009; 25: 2078–2079. 10.1093/bioinformatics/btp352 19505943 PMC2723002

[28] McKenna A, Hanna M, Banks E, Sivachenko A, Cibulskis K, Andrew K, et al The genome analysis toolkit: a MapReduce framework for analyzing next-generation DNA sequencing data. Genome Res. 2010; 20: 1297–1303. 10.1101/gr.107524.110 20644199 PMC2928508

[29] Katoh K, Misawa K, Kuma K, Miyata T. MAFFT: a novel method for rapid multiple sequence alignment based on fast Fourier transform. Nucleic Acids Res. 2002; 30: 3059–3066. 12136088 10.1093/nar/gkf436 PMC135756

[30] Hall TA. BIOEDIT: A user-friendly biological sequence alignment editor and analysis program for windows 95/98/NT. Nucleic Acids Symp Ser. 1999; 41: 95–98.

[31] Schmidt HA, Strimmer K, Vingron M, von Haeseler A. TREE-PUZZLE: maximum likelihood phylogenetic analysis using quartets and parallel computing. Bioinformatics. 2002; 18: 502–504. 11934758 10.1093/bioinformatics/18.3.502

[32] Posada D, Crandall KA. Modeltest: testing the model of DNA substitution. Bioinformatics. 1998; 14: 817–818. 9918953 10.1093/bioinformatics/14.9.817

[33] Swofford DL. PAUP: Phylogenetic analysis using parsimony. Ver. 4.0b10. Sunderland, Massachusetts: Sinauer Associates; 2003.

[34] Suyama M, Torrents D, Bork P. PAL2NAL: robust conversion of protein sequence alignments into the corresponding codon alignments. Nucleic Acids Res. 2006 34: W609–W612. 10.1093/nar/gkl315 16845082 PMC1538804

[35] Yang Z. PAML 4: phylogenetic analysis by maximum likelihood. Mol Biol Evol. 2007; 24: 1586–1591 10.1093/molbev/msm088 17483113

[36] Ronquist F, Huelsenbeck JP. MrBayes 3: Bayesian phylogenetic inference under mixed models. Bioinformatics. 2003; 19: 1572–1574. 12912839 10.1093/bioinformatics/btg180

[37] Guindon S, Gascuel O. A simple, fast and accurate algorithm to estimate large phylogenies by maximum likelihood. Syst Biol. 2003; 52: 696–704. 14530136 10.1080/10635150390235520

[38] Drummond AJ, Rambaut A. BEAST: Bayesian evolutionary analysis by sampling trees. BMC Evol Biol. 2007; 7: 214 10.1186/1471-2148-7-214 17996036 PMC2247476

[39] Forstén A. Mitochondrial-DNA time-table and the evolution of Equus: Comparison of molecular and paleontological evidence. Ann Zool Fenn. 1992; 28: 301–309.

[40] Suchard MA, Weiss RE, Sinsheimer JS. Bayesian selection of continuous- time Markov chain evolutionary models. Mol Biol Evo. 2001; l 18: 1001–1013. 10.1093/oxfordjournals.molbev.a003872 11371589

[41] Rambaut A. Available at http://evolve.zoo.ox.ac.uk/beast; 2012.

[42] Landry D. Noble brutes: how eastern horses transformed English culture. Baltimore, MD: Johns Hopkins University Press; 2008.

[43] Wallace J. The horse of America in his derivation, history and development. New York, NY: Wallace; 1897.

[44] Wentworth L. Thoroughbred racing stock. London, UK: Geo Allen and Unwin; 1960.

[45] McGahern A, Bower M, Edwards C, Brophy PO, Sulimova G, Zakharov I, et al Evidence for biogeographical patterning in mitochondrial DNA sequences in eastern horse populations. Anim Genet. 2006; 37: 494–497. 10.1111/j.1365-2052.2006.01495.x 16978180

[46] Prystupa J, Hind P, Cothran E, Plante Y. Maternal lineages in native Canadian equine populations and their relationship to the Nordic and Mountain and Moorland pony breeds. J Hered. 2012; 103: 380–390. 10.1093/jhered/ess003 22504109

[47] Winton CL, Hegarty MJ, McMahon R, Slavov GT, McEwan NR. Genetic diversity and phylogenetic analysis of native mountain ponies of Britain and Ireland reveals a novel rare population. Ecol Evol. 2013; 3: 934–947. 10.1002/ece3.507 23610635 PMC3631405

[48] Warmuth V, Campana M, Eriksson A, Bower M, Barker G, Manica A. Ancient trade routes shaped the genetic structure of horses in eastern Eurasia. Mol Ecol. 2013; 22: 5340–5351. 10.1111/mec.12491 24118338

[49] d’Andrade F. A short history of the Spanish horse and of the Iberian ‘‘Gineta” horsemanship for which this horse is adapted (Lisbon). 1973 Oficinas de S. José, Lisboa.

[50] Ludwig A, Pruvost M, Reissmann M, Benecke N, Brockmann GA, Castanos P, et al Coat color variation at the beginning of horse domestication. Science. 2009; 324: 485 10.1126/science.1172750 19390039 PMC5102060

[51] Anthony DW. The horse, the wheel, and language: How Bronze-Age riders from the Eurasian steppes shaped the modern world 2007 Princeton: Princeton University Press.

[52] Goldman N, Yang Z. A codon-based model of nucleotide substitution for protein-coding DNA sequences. Mol Biol and Evol. 1994; 11: 725–736. 7968486 10.1093/oxfordjournals.molbev.a040153

[53] Ning T, Li J, Lin K, Xiao H, Wylie S, Hua S, et al Complex evolutionary patterns revealed by mitochondrial genomes of the domestic horse. Curr Mol Med. 2014 14: 1286–1298. 25470284 10.2174/1566524014666141203100940

