# Supplementary material for: Origin and spread of Thoroughbred racehorses inferred from complete mitochondrial genome sequences: Phylogenomic and Bayesian coalescent perspectives
Source: PLoS One. 2018 Sep 14;13(9):e0203917. doi: 10.1371/journal.pone.0203917 (PMC6138400; doi:10.1371/journal.pone.0203917)
Supplement: S1 Table — (DOC) [file pone.0203917.s001.doc]

Table S1. 167 horse isolates (68 breeds) used in this study.

| **ID** | **Group** | **Breed** | **Country** | **Accession no.** |
| --- | --- | --- | --- | --- |
| 1. AkT01 | 5 | Akhal-Teke | Central Asia | JN398450 |
| 1. AkT02 | 5 | Akhal-Teke | Central Asia | JN398449 |
| 1. AkT03 | 5 | Akhal-Teke | Central Asia | JN398453 |
| 1. AkT04 | 5 | Akhal-Teke | Central Asia | JN398452 |
| 1. AkT05 | 5 | Akhal-Teke | Central Asia | JN398435 |
| 1. AkT06 | 2 | Akhal-Teke | Central Asia | JN398404 |
| 1. AkT07 | 2 | Akhal-Teke | Central Asia | JN398410 |
| 1. AkT08 | 4 | Akhal-Teke | Central Asia | JN398424 |
| 1. AkT09 | 4 | Akhal-Teke | Central Asia | JN398422 |
| 1. AkT10 | 1 | Akhal-Teke | Central Asia | JN398385 |
| 1. AkT11 | 1 | Akhal-Teke | Central Asia | JN398393 |
| 1. AkT12 | 1 | Akhal-Teke | Central Asia | HQ439441 |
| 1. AkT13 | 5 | Akhal-Teke | Central Asia | HQ439442 |
| 1. Alt01 | 2 | Altai | Central Asia | HQ439443 |
| 1. Alt02 | 5 | Altai | Central Asia | HQ439444 |
| 1. AmP01 | 4 | American Paint | North America | JN398421 |
| 1. And01 | 4 | Andalusian | South Europe | JN398430 |
| 1. And02 | 5 | Andalusian | South Europe | JN398443 |
| 1. App01 | 4 | Appaloosa | North America | HQ439446 |
| 1. Arb01 | 5 | Arabian | Middle East | JN398448 |
| 1. Arb02 | 1 | Arabian | Middle East | JN398392 |
| 1. Arb03 | 2 | Arabian | Middle East | JN398406 |
| 1. Arb04 | 4 | Arabian | Middle East | JN398434 |
| 1. Arb05 | 1 | Arabian | Middle East | JN398380 |
| 1. Arb06 | 2 | Arabian | Middle East | JN398412 |
| 1. Arb07 | 5 | Arabian | Middle East | HQ439447 |
| 1. Arb08 | 5 | Arabian | Middle East | HQ439448 |
| 1. Arb09 | 1 | Arabian | Middle East | HQ439449 |
| 1. Ard01 | 5 | Ardennais | West Europe | HQ439450 |
| 1. BaC01 | 4 | Bashkir Curly | North America | HQ439451 |
| 1. BaC02 | 4 | Bashkir Curly | North America | HQ439452 |
| 1. Bar01 | 4 | Barb | North Africa | HQ439453 |
| 1. BeD01 | 3 | Belgian Draft | Central Europe | JN398420 |
| 1. BlF01 | 1 | Black Forest | Central Europe | HQ439487 |
| 1. Cam01 | 2 | Camargue | West Europe | HQ439454 |
| 1. ChP01 | 1 | Chincoteague Pony | North America | JN398377 |
| 1. Cly01 | 5 | Clydesdale | North Europe | JN398439 |
| 1. Cly02 | 5 | Clydesdale | West Europe | HQ439455 |
| 1. CsP01 | 1 | Caspian Pony | Middle East | JN398378 |
| 1. CsP02 | 4 | Caspian Pony | Middle East | JN398428 |
| 1. CsP03 | 5 | Caspian Pony | Middle East | JN398447 |
| 1. CsP04 | 2 | Caspian Pony | Middle East | JN398416 |
| 1. CsP05 | 5 | Caspian Pony | Middle East | JN398436 |
| 1. Deq01 | 5 | Deqin | Central Asia | EF597514 |
| 1. EnS01 | 5 | English Shire | North Europe | JN398440 |
| 1. ExP01 | 5 | Exmoor Pony | North Europe | JN398442 |
| 1. Fre01 | 5 | Friesian | Central Europe | JN398438 |
| 1. Gia01 | 2 | Giara (Sardinia) | South Europe | JN398411 |
| 1. Gia02 | 2 | Giara (Sardinia) | South Europe | JN398407 |
| 1. GRP01 | 4 | German Riding Pony | Central Europe | HQ439461 |
| 1. GSH01 | 4 | German Sport Horse | Central Europe | HQ439456 |
| 1. Haf01 | 5 | Haflinger | Central Europe | HQ439464 |
| 1. Han01 | 5 | Hanoverian | Central Europe | HQ439457 |
| 1. Hol01 | 1 | Holstein | Central Europe | HQ439458 |
| 1. HuC01 | 4 | Hungarian Coldblood | Central Europe | HQ439494 |
| 1. IcH01 | 1 | Icelandic Horse | North Europe | JN398400 |
| 1. IcH02 | 1 | Icelandic Horse | North Europe | JN398399 |
| 1. IcH03 | 5 | Icelandic Horse | North Europe | HQ439465 |
| 1. IcH04 | 5 | Icelandic Horse | North Europe | HQ439466 |
| 1. Irn01 | 4 | Unspecified Iranian | Middle East | JN398423 |
| 1. Irn02 | 3 | Unspecified Iranian | Middle East | JN398419 |
| 1. Irn03 | 5 | Unspecified Iranian | Middle East | JN398446 |
| 1. Irn04 | 6 | Unspecified Iranian | Middle East | JN398457 |
| 1. Irn05 | 2 | Unspecified Iranian | Middle East | JN398405 |
| 1. Irn06 | 5 | Unspecified Iranian | Middle East | JN398444 |
| 1. Irn07 | 5 | Unspecified Iranian | Middle East | JN398451 |
| 1. Irn08 | 5 | Unspecified Iranian | Middle East | JN398455 |
| 1. Irn09 | 1 | Unspecified Iranian | Middle East | JN398395 |
| 1. Irn10 | 2 | Unspecified Iranian | Middle East | JN398415 |
| 1. Irn11 | 2 | Unspecified Iranian | Middle East | JN398414 |
| 1. Irn12 | 5 | Unspecified Iranian | Middle East | JN398445 |
| 1. Irn13 | 4 | Unspecified Iranian | Middle East | JN398433 |
| 1. Irn14 | 1 | Unspecified Iranian | Middle East | JN398383 |
| 1. Ita01 | 1 | Unspecified Italian | South Europe | JN398390 |
| 1. Ita02 | 2 | Unspecified Italian | South Europe | JN398409 |
| 1. Ita03 | 4 | Unspecified Italian | South Europe | JN398425 |
| 1. Jeju01 | 5 | Jeju | Korea (Jeju) | KF038159 |
| 1. Jeju02 | 5 | Jeju | Korea (Jeju) | KF038160 |
| 1. Jeju03 | 2 | Jeju | Korea (Jeju) | KF038161 |
| 1. Jeju04 | 1 | Jeju | Korea (Jeju) | KF038162 |
| 1. Jeju05 | 3 | Jeju | Korea (Jeju) | KF038163 |
| 1. Jeju06 | 1 | Jeju | Korea (Jeju) | KF038164 |
| 1. Kab01 | 2 | Kabardin | East Europe | HQ439468 |
| 1. KiH01 | 1 | KinskyHorse | Central Europe | HQ439469 |
| 1. KiH02 | 4 | Kinsky Horse | Central Europe | HQ439470 |
| 1. Kla01 | 5 | Kladruber | Central Europe | HQ439445 |
| 1. Kla02 | 1 | Kladruber | Central Europe | HQ439471 |
| 1. Kla03 | 2 | Kladruber | Central Europe | HQ439472 |
| 1. Kon01 | 4 | Konik | Central Europe | HQ439473 |
| 1. Kus01 | 2 | Kustanai | Central Asia | HQ439476 |
| 1. Kuz01 | 1 | Kuznet | East Europe | HQ439474 |
| 1. Kuz02 | 1 | Kuznet | East Europe | HQ439475 |
| 1. Lew01 | 5 | Lewitzer | Central Europe | HQ439477 |
| 1. Lie01 | 4 | Liebenthaler | Central Europe | HQ439478 |
| 1. Lie02 | 2 | Liebenthaler | Central Europe | HQ439479 |
| 1. Lus01 | 1 | Lusitano | Portugal | X79547 |
| 1. Mrm01 | 5 | Maremmano | South Europe | JN398437 |
| 1. Mrm02 | 5 | Maremmano | South Europe | JN398454 |
| 1. Mrm03 | 4 | Maremmano | South Europe | JN398427 |
| 1. Mrm04 | 4 | Maremmano | South Europe | JN398432 |
| 1. Mrm05 | 2 | Maremmano | South Europe | JN398401 |
| 1. Mrm06 | 2 | Maremmano | South Europe | JN398413 |
| 1. Mrm07 | 1 | Maremmano | South Europe | JN398382 |
| 1. Mrm08 | 1 | Maremmano | South Europe | JN398388 |
| 1. Mrm09 | 6 | Maremmano | South Europe | JN398456 |
| 1. Mrm10 | 4 | Maremmano | South Europe | JN398426 |
| 1. Mrm11 | 1 | Maremmano | South Europe | JN398381 |
| 1. Mrm12 | 1 | Maremmano | South Europe | JN398379 |
| 1. Mrm13 | 1 | Maremmano | South Europe | JN398397 |
| 1. Mrm14 | 1 | Maremmano | South Europe | JN398387 |
| 1. Mrm15 | 3 | Maremmano | South Europe | JN398418 |
| 1. Mon01 | 4 | Mongolian | Mongol | KF038165 |
| 1. Mon02 | 4 | Mongolian | Mongol | KF038166 |
| 1. Naq01 | 2 | Naqu | Central Asia | EF597513 |
| 1. NoF01 | 1 | Norwegian Fjord | North Europe | JN398398 |
| 1. NoF02 | 2 | Norwegian Fjord | North Europe | HQ439463 |
| 1. Nor01 | 2 | Noriker | Central Europe | HQ439480 |
| 1. Old01 | 4 | Oldenburg | North Europe | JN398429 |
| 1. Old02 | 4 | Oldenburg | Central Europe | HQ439459 |
| 1. OrT01 | 2 | Orlov Trotter | East Europe | HQ439481 |
| 1. PaH01 | 4 | Painted Horse | North America | HQ439482 |
| 1. Per01 | 4 | Percheron | West Europe | HQ439483 |
| 1. Prz01 | 2 | Przewalskii | Central Asia | JN398402 |
| 1. Prz02 | 2 | Przewalskii | East Asia | HQ439484 |
| 1. *PrzK01 | 2 | Przewalskii | Mongol | KT221844 |
| 1. *PrzK02 | 2 | Przewalskii | Mongol | KT221845 |
| 1. RHD01 | 1 | Rhineland Heavy Draft | Central Europe | HQ439491 |
| 1. RHD02 | 5 | Rhineland Heavy Draft | West Europe | HQ439485 |
| 1. RRH01 | 1 | Russian Riding Horse | East Europe | HQ439486 |
| 1. Sad01 | 5 | Saddlebred | North America | JN398441 |
| 1. ShA01 | 1 | Shagya Arab | Central Europe | HQ439488 |
| 1. She01 | 1 | Shetland | West Europe | HQ439489 |
| 1. Shi01 | 2 | Shire | West Europe | HQ439490 |
| 1. Sil01 | 4 | Silesian | Central Europe | JN398431 |
| 1. SpH01 | 4 | Spotted Horse | Central Europe | HQ439492 |
| 1. SuP01 | 1 | Suffolk Punch | North Europe | JN398396 |
| 1. Syr01 | 1 | Unspecified Syrian | Middle East | JN398394 |
| 1. Syr02 | 1 | Unspecified Syrian | Middle East | JN398384 |
| 1. Syr03 | 2 | Unspecified Syrian | Middle East | JN398408 |
| 1. Syr04 | 1 | Unspecified Syrian | Middle East | JN398391 |
| 1. Syr05 | 1 | Unspecified Syrian | Middle East | JN398389 |
| 1. Thor01 | 1 | Thoroughbred | West Europe | HQ439462 |
| 1. *ThorK01 | 4 | Thoroughbred | South Korea | KT221830 |
| 1. *ThorK02 | 2 | Thoroughbred | South Korea | KT221831 |
| 1. *ThorK03 | 2 | Thoroughbred | South Korea | KT221832 |
| 1. *ThorK04 | 4 | Thoroughbred | South Korea | KT221833 |
| 1. *ThorK05 | 2 | Thoroughbred | South Korea | KT221834 |
| 1. *ThorK06 | 4 | Thoroughbred | South Korea | KT221835 |
| 1. *ThorK07 | 2 | Thoroughbred | South Korea | KT221836 |
| 1. *ThorK08 | 1 | Thoroughbred | South Korea | KT221837 |
| 1. *ThorK09 | 5 | Thoroughbred | South Korea | KT221838 |
| 1. *ThorK10 | 2 | Thoroughbred | South Korea | KT221839 |
| 1. *ThorK11 | 4 | Thoroughbred | South Korea | KT221840 |
| 1. *ThorK12 | 1 | Thoroughbred | South Korea | KT221841 |
| 1. *ThorK13 | 1 | Thoroughbred | South Korea | KT221842 |
| 1. *ThorK14 | 4 | Thoroughbred | South Korea | KT221843 |
| 1. Tra01 | 2 | Trakhener | North Europe | JN398417 |
| 1. Tra02 | 4 | Trakehner | Central Europe | HQ439493 |
| 1. VHD01 | 5 | Vladimir Heavy Draught | East Europe | HQ439500 |
| 1. Via01 | 5 | Viatka | East Europe | HQ439495 |
| 1. WeC01 | 2 | Welsh Cob | West Europe | HQ439496 |
| 1. WeP01 | 2 | Welsh Pony | West Europe | HQ439497 |
| 1. WeP02 | 4 | Welsh Pony | West Europe | HQ439498 |
| 1. WeP03 | 2 | Welsh Pony | West Europe | HQ439499 |
| 1. Wes01 | 1 | Westphalian | Central Europe | JN398386 |
| 1. Wes02 | 1 | Westphalian | Central Europe | HQ439460 |
| 1. Yak01 | 5 | Yakut | East Asia | HQ439467 |
| 1. Donkey |  | *Equus asinus* (outgroup) | North Europe? | X97337 |

Note: New sequences marked with asterisk.
