# Supplementary material for: Origin and spread of Thoroughbred racehorses inferred from complete mitochondrial genome sequences: Phylogenomic and Bayesian coalescent perspectives
Source: PLoS One. 2018 Sep 14;13(9):e0203917. doi: 10.1371/journal.pone.0203917 (PMC6138400; doi:10.1371/journal.pone.0203917)
Supplement: S2 Table — (DOCX) [file pone.0203917.s002.docx]

Table S2. Summary statistics for number of reads used in this study and alignment rate using SAMtools.

| Sample name | Read length | Number of total reads | Remained read after filtering | Mapped reads | Properly paired reads | With itself and mate mapped reads | Singletons |
| --- | --- | --- | --- | --- | --- | --- | --- |
| ThorK01 | 104 | 442,468,340 | 442,453,615 | 62,305 (0.01%) | 61,462 (0.01%) | 61,918 (0.01%) | 387 (0.00%) |
| ThorK02 | 104 | 451,433,906 | 451,412,702 | 42,436 (0.01%) | 41,568 (0.01%) | 41,978 (0.01%) | 458 (0.00%) |
| ThorK03 | 104 | 459,872,496 | 459,850,980 | 40,629 (0.01%) | 39,804 (0.01%) | 40,124 (0.01%) | 505 (0.00%) |
| ThorK04 | 104 | 447,573,054 | 447,557,178 | 51,297 (0.01%) | 50,392 (0.01%) | 50,772 (0.01%) | 525 (0.00%) |
| ThorK05 | 104 | 440,002,798 | 439,983,677 | 51,982 (0.01%) | 51,114 (0.01%) | 51,540 (0.01%) | 442 (0.00%) |
| ThorK06 | 104 | 490,484,242 | 490,457,929 | 56,732 (0.01%) | 55,756 (0.01%) | 56,320 (0.01%) | 412 (0.00%) |
| ThorK07 | 104 | 511,439,430 | 511,419,560 | 35,652 (0.01%) | 34,724 (0.01%) | 35,056 (0.01%) | 596 (0.00%) |
| ThorK08 | 104 | 407,375,444 | 407,354,534 | 57,854 (0.01%) | 56,970 (0.01%) | 57,480 (0.01%) | 374 (0.00%) |
| ThorK09 | 104 | 417,011,498 | 416,990,438 | 45,454 (0.01%) | 44,602 (0.01%) | 45,036 (0.01%) | 418 (0.00%) |
| ThorK10 | 104 | 401,775,304 | 401,768,298 | 56,182 (0.01%) | 55,332 (0.01%) | 55,734 (0.01%) | 448 (0.00%) |
| ThorK11 | 104 | 493,236,516 | 493,214,730 | 41,015 (0.01%) | 40,158 (0.01%) | 40,492 (0.01%) | 523 (0.00%) |
| ThorK12 | 104 | 508,102,058 | 508,080,010 | 73,608 (0.01%) | 72,544 (0.01%) | 73,234 (0.01%) | 374 (0.00%) |
| ThorK13 | 104 | 488,414,810 | 488,404,507 | 70,443 (0.01%) | 69,616 (0.01%) | 70,142 (0.01%) | 301 (0.00%) |
| ThorK14 | 104 | 481,422,916 | 481,416,327 | 70,598 (0.01%) | 69,646 (0.01%) | 70,222 (0.01%) | 376 (0.00%) |
| PrzK01 | 99 | 661,815,536 | 661,815,384 | 48,376 (0.01%) | 42,728 (0.01%) | 43,440 (0.01%) | 4,936 (0.00%) |
| PrzK02 | 99 | 735,775,306 | 735,774,944 | 72,923 (0.01%) | 69,716 (0.01%) | 70,886 (0.01%) | 2,037 (0.00%) |
