# Supplementary material for: Origin and spread of Thoroughbred racehorses inferred from complete mitochondrial genome sequences: Phylogenomic and Bayesian coalescent perspectives
Source: PLoS One. 2018 Sep 14;13(9):e0203917. doi: 10.1371/journal.pone.0203917 (PMC6138400; doi:10.1371/journal.pone.0203917)
Supplement: S3 Table — (DOCX) [file pone.0203917.s003.docx]

Table S3. Number of variants (SNPs and InDels) identified using GATK.

| Sample name | SNP | Indel |
| --- | --- | --- |
| ThorK01 | 82 | 24 |
| ThorK02 | 73 | 15 |
| ThorK03 | 72 | 27 |
| ThorK04 | 83 | 22 |
| ThorK05 | 72 | 16 |
| ThorK06 | 80 | 27 |
| ThorK07 | 74 | 15 |
| ThorK08 | 30 | 14 |
| ThorK09 | 124 | 15 |
| ThorK10 | 70 | 12 |
| ThorK11 | 82 | 9 |
| ThorK12 | 79 | 10 |
| ThorK13 | 53 | 7 |
| ThorK14 | 81 | 11 |
| PrzK01 | 96 | 12 |
| PrzK02 | 95 | 13 |
