# Supplementary material for: Origin and spread of Thoroughbred racehorses inferred from complete mitochondrial genome sequences: Phylogenomic and Bayesian coalescent perspectives
Source: PLoS One. 2018 Sep 14;13(9):e0203917. doi: 10.1371/journal.pone.0203917 (PMC6138400; doi:10.1371/journal.pone.0203917)
Supplement: S4 Table — (DOCX) [file pone.0203917.s004.docx]

Table S4. tMRCA of Thoroughbred horses (My). Estimates for Thoroughbred horses ranged from 8,100 years (ThorK14) to 111,500 years (Thor01).

| Sample name | tMRCA | 95% HPD interval |
| --- | --- | --- |
| Thor01 | 0.1115 | 0.0257 – 0.2338 |
| ThorK01 | 0.0102 | 0.009 – 0.0898 |
| ThorK02 | 0.0575 | 0.0098 – 0.156 |
| ThorK03 | 0.0312 | 0.0021 – 0.0919 |
| ThorK04 | 0.0102 | 0.0004 – 0.0314 |
| ThorK05 | 0.0637 | 0.0295 – 0.1847 |
| ThorK06 | 0.0081 | 0.0002 – 0.0277 |
| ThorK07 | 0.0685 | 0.0151 – 0.1472 |
| ThorK08 | 0.0336 | 0.0043 – 0.0905 |
| ThorK09 | 0.0178 | 0.0025 – 0.051 |
| ThorK10 | 0.0443 | 0.0062 – 0.1269 |
| ThorK11 | 0.0526 | 0.0085 – 0.1312 |
| ThorK12 | 0.0403 | 0.0287 – 0.161 |
| ThorK13 | 0.0237 | 0.001 – 0.0751 |
| ThorK14 | 0.0081 | 0.0002 – 0.0277 |
